# Bioplastics: A new analytical challenge

**DOI:** 10.3389/fchem.2022.971792

**Published:** 2022-09-23

**Authors:** Valentina Censi, Filippo Saiano, David Bongiorno, Serena Indelicato, Anna Napoli, Daniela Piazzese

**Affiliations:** ^1^ Department of Earth and Marine Sciences, University of Palermo, Palermo, Italy; ^2^ Department Agricultural Food and Forestry Sciences, University of Palermo, Palermo, Italy; ^3^ Department of Biological, Chemical and Pharmaceutical Science and Technology (STEBICEF), University of Palermo, Palermo, Italy; ^4^ Department of Chemistry and Chemical Technologies, University of Calabria, Arcavacata di Rende (CS), Italy

**Keywords:** bio-plastics, bio-polymers determination, analytical, methods, mass spectrometry, bioplastic degradation

## Abstract

Even though petroleum-based plastics are advantageous in complying with the performance requirements in many applications, these are related, throughout their life cycle, to several environmental problems, including greenhouse gas emissions and persistence in marine and terrestrial environments. Therefore, the preservation of natural resources and climate change is considered worldwide, the main reason for which is necessary to reduce consumption and dependence on fossil-based materials. Biopolymers (PLA, PHAs, etc.) are examples of plastics whose use is grown exponentially over the years because of the improvements of their physical and mechanical properties using additives of various nature and depending on the scope of application. This review aims to discuss various ways of biopolymer degradation, to evaluate if they represent a new Frontier in eco-sustainability or rather a re-proposal of old problems. Related to this topic, we also have focussed our attention on the different methods for the quantitative analysis of bioplastics, or their degradation by-products, comparing and evaluating the advantages and disadvantages of each technique.

## GRAPHICAL ABSTRACT

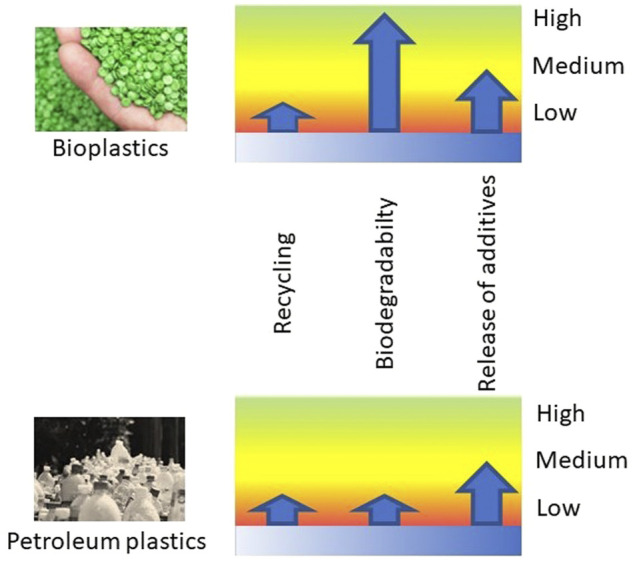

## 1 Introduction

Plastics are light, strong durable, and inexpensive synthetic or semi-synthetic organic polymers. Their characteristics justify the very large exploitation in a wide spectrum of human activities explaining why plastics represent an essential element in modern life. About 99% of plastics are made starting from non-renewable resources such as charcoal, petroleum, and natural gas. Accordingly, about 20% of the overall petroleum consumption could be related to the plastics industry (Kasavan 2021). In Europe, plastics are mainly exploited in the packaging (∼40%), construction field (∼20%), textile industry (∼15%), automotive (∼10%), and goods (∼10%). Considering all these applications, the use of plastics could grow in the next future (World Economic Forum 2016; Gu 2017).

Even if the use of plastics has several benefits, their environmental occurrence is currently a very dangerous issue (Millican 2021) due to the progressive abrasion until the particulate formation. This is usually classified according to dimensions as nano-plastics (ø < 10–4 mm), microplastics (ø < 5 mm), mesoplastics (5 < ø < 25 mm) and macroplastics (ø > 25 mm). Proper management of plastic wastes would be hoped since the abrasion of these solids provides large amounts of plastic particles dangerous for organisms and humans (Filho 2019; Zhao 2021). This mainly occurs in marine systems where plastic wastes are accumulated at the end of their environmental path. Here, the characteristics of durability and resistance of plastics become a great problem in non-correct plastic disposal (Rosenboom 2022; Filho 2021). For these reasons, the critical review work was carried out by selecting data and information mainly related to the degradation process of biopolymers and their potential environmental impact. The knowledge of these materials in the production phase has now reached very good levels of completeness, as evidenced by the huge of information already present in the literature. The analytical-environmental challenge remains open, as highlighted by the insufficient information available for the assessment of the sustainability of biopolymers and their degradation products.

## 2 Bioplastics

As often reported in the literature, the term “bioplastics” define materials produced from biomass sources (vegetable fats and oils, corn starch, straw, woodchips, sawdust, etc.) and recycled food waste, which can be divided into biodegradable, bio-based, or both. Biodegradable bioplastics are degraded in aqueous fluids under the effects of bacterial activity. These processes lead, as the last step, to CO_2_ and H_2_O formation in aerobic degradation and CO_2_ and CH_4_ formation in anaerobic degradation. Bio-based bioplastics in whole or in part result from biomass-based syntheses (Di Bartolo 2021; Naser 2021). Some examples of bioplastics are poly (3-hydroxybutyrate) (PHB), poly (ε-caprolactone) (PCL), poly (butylene succinate) (PBS), poly (lactic acid) (PLA), and poly (ethylene succinate) (PES) (Atiwesh 2021, Roohi 2018). According to their versatility and their capacity to satisfy international standards of certification of composting (Naser 2021) and marine biodegradation (Meereboer 2020), respectively, poly-hydroxy-alkenoates (PHAs) and poly (lactic acid) (PLA) are among the most widespread biopolymers and together with polypropylene show the highest relative growth rate in their industrial production (Naser 2021).

Poly-hydroxy-alkanoates (PHAs) are non-toxic biocompatible aliphatic bio-polyesters, from microbial fermentation of renewable sources (Venkatachalam 2020; Suzuki 2021). PHAs can be produced from raw materials in wastewater treatment plants, landfills, composting facilities, and farms (Atiwesh 2021). PHAs can also come from wood chips, grass, and green wastes rather than from the more expensive edible crop biomass. PHAs are flexible and crystalline with similar thermoplastic and mechanical properties to synthetic plastics (Akinmulewo 2019). PHAs’ properties can change according to the arrangement of different monomers forming the polymer structure, or working on bacterial colonies, fermentation, or substratum. In this way, differing PHA well suited for packaging, fibers, and medical implants were developed differently combining polyesters groups and radical chains.

PHAs can be classified according to the polymer length: long (more than 14 C atoms), medium (between 6 and 14 C atoms), and short chain (between 3 and 5 C atoms) (Albuquerque 2018; Muniyandi 2020).

Poly (3-hydroxybutyrate) (PHB) is the most studied among PHA. It represents the C stock of several bacterial colonies produced through bacterial fermentation from CH_4_, which is first oxidized to methanol, via the methane monooxygenase enzyme catalytic pathway, in the second step methanol is transformed into formaldehyde by methanol dehydrogenase (Atiwesh 2021). PHB shows a linear structure as a sequence of CH_3_ and CH_2_, followed by an ester -COOR group that results in physical-chemical (thermoplasticity, hydrophobicity) and mechanic properties (crystallinity grade and fragility). Commercial PHB shows similar properties to polypropylene (PP) coming from fossil fuels, showing significant stiffness, fragility, a crystallinity grade between 60 and 80%, a fusion temperature close to 180°C, and both an amorphous and a crystalline phase (Suzuki 2021). PHB can be a virgin polymer or with copolymers and additives in blends with better thermoplastic properties, such as the poly (3-hydroxybutyrate-*co*-3-hydroxyvalerate) [P (3HB-*co*-HV)] (McAdam 2020). Poly (lactic acid) (PLA) is both a bio-based and biodegradable thermoplastic polymer belonging to the aliphatic poly-esters class resulting from *α*-hydroxy acids. It is synthesized from lactic acid through bacterial fermentation of renewable plant-based sources or polymerizing the cyclic lactide dimer after a ring-opening reaction (Ring-Opening Polymerization, ROP) (Masutani 2014). PLA, for commercial purposes, is among the most promising bioplastics due to its mechanical properties, processability, renewability, and non-toxicity (Atiwesh 2021). PLA has larger durability than most biodegradable polymers, with larger transparency and mechanical strength. These properties and their widespread exploitation in several fields make ever-growing worldwide PLA production (Jiménez 2019).

### 2.1 Physico-chemical characterization

The structural and physicochemical properties of the bioplastics both in solid and solution phases have been extensively studied employing varying analytical approaches intended to characterize their chemical-physical features from several points of view. Thermal techniques such as Thermogravimetric analysis - TGA, and Differential Scanning Calorimetry–DSC, have been used to determine the material properties temperature-dependent in the bulk (Vahabi 2019). Spectroscopic techniques such as FT-IR, UV-Vis, and Fluorescence, are mostly used to highlight variations in the building blocks of the polymer structure (Han 2016). Spectrometric approaches such as NMR and mass-spectrometry are widely used for the qualitative and quantitative determination of several classes of substances including low molecular weight pollutants, high molecular weight polymers up to supramolecular aggregates (Banerjee 2012; Indelicato 2016; Biale 2021; Krawczyk-Walach 2021) Therefore, all these analytical approaches can be very informative in synergy when it comes to evaluating a material undergoing various stresses or degradative processes (Kumar 2017; Akdoğan 2018; Falkenstain 2021; Ghasemlou 2022).

### 2.2 Benefits and drawbacks

The main benefit of bioplastic exploitation comes from its production from renewable sources. However, biomass production requires suitable spaces, large water consumption, and intensive farming finalized to increase production. Accordingly, bioplastic production can involve the use of pesticides in crops and chemicals during the transformation processes that could be avoided by resorting to eco-friendly syntheses (Arikan 2015). On the other hand, bioplastics are biodegradable without any filler addition, which is often used for increasing their mechanical properties (Iwata 2015; Abe 2021; Van Roijen 2022). Despite the growing request for bioplastics, some handicaps to their larger exploitation come from their expensive production and recycling (Chen 2014; Coppola 2021; Nandakumar 2021; Shah 2021). A cost reduction could be provided from the PHA extraction from cells through halogenated solvents such as CHCl_3_ and CH_2_Cl_2_. But these are toxic chemicals that would be largely used for avoiding too viscous polymeric solutions. Further industrial methods for PHA recovery were attempted but a limited PHA yield was obtained (Yang 2015).

## 3 Degradation process

The study of the degradation process is useful for assessing the environmental impact of bioplastic waste and finding an appropriate measure for implementing waste legislation and policies (Nandakumar 2021; Ghasemlou 2022).

Biodegradation is the process by which materials can be decomposed by microorganisms and used as a food source. The final products of the biodegradation process are CO_2_ and H_2_O, as also biomass and methane. However, although the material is biodegradable, it may not be in all circumstances or conditions. Several factors influence the biodegradation process, which adds up to microbial density and environmental conditions (i.e., temperature, humidity). These factors are polymer composition, molecular weight, crystallinity, pH, chemical structure, morphology, hydrophilicity, and breakdown products, but the relative extent of their effects is unclear (Meereboer 2020).

The first stage involves the enzymatic or chemical hydrolysis of the polymer chain and the consequent formation of degradation products whose size allows for microorganisms’ encapsulation. It follows the decomposition and bio assimilation of the fragmented polymers up to their conversion into carbon dioxide, nitrogen oxide, methane, and water. For this reason, several international methods for assessing the biodegradability of plastics are based on the quantification of carbon dioxide production or the biochemical oxygen demand during the decomposition process (Suzuki 2021).

The abiotic or chemical degradation of bioplastics involves various techniques: pyrolysis, hydrolysis, alcoholysis, and glycolysis. Pyrolysis is a process of thermal cracking, in which the polymer, by heating in an inert environment, is converted into organic vapors, carbons, and gases. In the second stage, these by-products are converted into oil through a condensation process.

The processes of hydrolysis, alcoholysis, and glycolysis are techniques of depolymerization in which the polymer chain is broken down by the action of the water, alcohol, or glycol respectively (Lamberti 2020). The material is hydrolyzed through mechanisms of mass or surface erosion. Surface erosion occurs when the rate of hydrolysis exceeds the rate of diffusion of water in the mass (Piemonte 2013). This mechanism represents the main degradation process for hydrophobic and semi-crystalline polymers and for polymers that exhibit a very rapid hydrolysis rate (Meereboer 2020). The hydrolysis mechanism can change from surface to mass erosion when the material reaches a threshold thickness. Conversely, if the diffusion of water is fast to the rate of hydrolysis, degradation occurs through the entire mass of the polymer leading to a homogeneous erosion. The rate of hydrolysis is affected by several external factors, for example, an increase in temperature promotes the rate of hydrolysis, and therefore a change in the pH value. Alcoholisys occurs when there is a transesterification reaction in which the alcohol group cleaves the external bonds, and the polymer chain splits into its monomers or oligomers. The glycolysis of polyesters involves the insertion of glycol in the polymer chains, breaking the external bonds and replacing them with hydroxyl terminals (Lamberti 2020).

The reason for the large commercial spread of biodegradable bioplastics lies in their easy degradation, which would allow, under natural conditions, in soil, water, and sediment, to rapidly reduce the amount of plastic waste eventually improperly disposed of. The factor limiting the degree of degradation of most bioplastics is the surface of the polymer both in the case of chemical or enzymatic degradation. As already mentioned, the polymer surface is a fundamental aspect in the study of the degradation processes. Biotic (enzymatic) degradation occurs on the surface due to the enzymes’ large size which prevents their permeation in the inner polymer structure. Enzymatic hydrolysis of biopolymers is a two-step process: degradation begins with the adsorption of enzymes on the polymer’s surface-active sites. The second step is a hydrolytic cleavage of polymer chain bonds, which is induced by the binding site of the hydrophobic portion and the catalytic site respectively (Meereboer 2020). Accelerated by enzymes, surface degradation is much faster, especially in soil, and over time causes an increase in surface and roughness of the biopolymer and consequently higher hydrophilicity (Meereboer 2020, Muthukumar 2015).

## 4 Analytical investigation

As bioplastics continue to enter the environment, understanding their qualitative and quantitative impact is vital to addressing their potential environmental pollution. Bioplastics can enter the environment as small particles, which can lead to a significant biological and toxicological impact (González-Pleiter 2019). In this regard, the ability to quantify and identify the nature of particles smaller than 10 μm has become a challenge. The following step, in the possible environmental pollution of bioplastics, is the production, due to the degradation process, of both solid microparticles and soluble compounds. These two ways are in the field of analytical sciences very complex challenges in the identification and quantification of bioplastic degradation products. For this reason, we will discuss in this paragraph the last analytical techniques used in the quali-quantitative analysis of bioplastic degradation products.

### 4.1 Qualitative studies

Qualitative analysis of building blocks in bioplastics is possible by spectroscopic methods such as fluorescence, nuclear magnetic resonance (NMR), Fourier-transform infrared spectroscopy (FTIR), or UV-VIS spectrophotometric techniques (Kumar 2017; Akdoğan 2018). Qualitative aspects of bioplastic degradation are of critical importance to planning, when possible, the following quantitative studies. Microparticles can be determined analytically either by analyzing them as they are or by analyzing them after dissolution in solvents. FT-IR micro imaging, gas chromatography with mass spectrometry detector, and thermal analysis represent the most used analytical techniques for the analysis of the solid debris of microparticles, while liquid chromatography, always with mass spectrometry detection, is currently the most widely used technique in the analysis of the dissolved microplastics or of their fractions (Sikorska 2020; Ye 2022; Yusuf 2022). Microparticles can be determined in their native status, or after dissolution in solvents. FT-IR micro imaging, gas chromatography coupled with mass spectrometry (GC-MS), and thermal analysis represent the most used analytical techniques for the investigation of solid microparticles. Liquid chromatography, coupled with mass spectrometry (LC-MS) detection, is currently widely used for dissolved microplastic fractions, (Sikorska 2020; Ye 2022; Yusuf 2022).

#### 4.1.1 Fourier transform infrared spectroscopy

FT-IR analysis has the advantage of being a non-destructive and rapid technique, with minimal sample preparation. The PHAs analysis is based on the study of the variation of the intensity of the stretching band of the carbonyl of PHAs as a function of concentration, in the range of 1728–1740 cm^−1^ of wave numbers (Godbole 2016; Isak 2016). However, in the last years, to investigate particles up to 10 μm, the more helpful instrument is the FT-IR micro imaging system, (μFT-IR Imaging). μFT-IR is an instrument with a state-of-the-art infrared detector that simultaneously generates a high number of spatially resolved spectra and analyses large sets of microplastic data showing a “visible” image of the sample. Through appropriate software, it compares the spectra of the microparticles present in environmental matrices with a database of the reference spectra, allowing a very good identification of the microplastic by size, volume, and mass (Karami 2018, Liebezeit 2014).

#### 4.1.2 UV-vis spectrophotometry

This classical and easy technique finds an interesting use in the determination of PHB by exploiting the degradation of the molecule of P (3HB) to crotonic acid by heating in concentrated sulphuric acid and determining its content by studying the absorbance mass of the crotonic acid band at 235 nm (Duvigneau 2021). This technique nowadays, for its easiness, is still used, though do not allow us to determine PHB copolymers.

#### 4.1.3 Fluorescence spectroscopy

The application of this technique in PHAs analysis is based on the fluorescence of Nile-Red, a lipid fluorochrome, that easily penetrates the suspended cells making fluorescent the polymer portion contained in them. The concentration of polymer in the cells can be determined from the analysis of the fluorescence intensity. The intensity of the fluorescence emission of red-stained cells with Nile-Red increases with the biopolymer concentration. This method for the determination of the biopolymer concentration has several advantages: it is fast and reproducible, measurements can be made immediately after sampling, sample preparation time is shorter than traditional methods and sample volumes for analytical determination are very small (Godbole 2016; Rajankar 2018).

However, Nile Red can stain not only biopolymers (PHAs or PLA) but also other lipophilic compounds (Arikawa 2017) and for this reason, fluorescence spectroscopy can be classified as a qualitative rather than quantitative technique.

#### 4.1.4 Qualitative thermal analysis

Among the different techniques available, thermal techniques are widely used in the design, preparation, and characterization of polymeric materials.

Thermal analysis (Differential Scanning Calorimetry and Thermogravimetric Analysis) offers, in addition to high precision in measurement, smart execution, allowing to obtain with a very limited amount of material valuable information regarding the property-structure correlation (Blanco 2021).

### 4.2 Quantitative studies

#### 4.2.1 Gas chromatography

Gas chromatography has the advantage of providing very detailed, accurate, reproducible, and precise measurements, but conversely uses solvents harmful to the environment and requests long sample pre-treatment and large quantities of the sample (Khok 2020).

The most common method used to determine the PHA content in cells is gas chromatography with a flame ionization detector (GC-FID). This method is quite laborious, but it has high accuracy and provides extensive information on the composition of the monomer of PHAs (Isak 2016).

GC-MS allows the determination of PHB content in cell biomass after an initial stage of methanolysis (Khok 2020), P (3HB) content after acidic or basic digestion, and PHA content from freeze-dried bacteria or natural sample extracts after acidic digestion (Godbole 2016).

The GC-MS drawback is the use of not eco-friendly solvents. To overcome this side effect, it is better to use the pyrolysis technique directly coupled with gas chromatography (Py-GC), which is a valid method in the direct analysis of the content of PHB and its copolymers in prokaryotes (Baidurah 2016; Khang 2021).

#### 4.2.2 Quantitative thermal analyses

Thermogravimetric analysis associated with mass spectrometry (TGA-MS) is a direct method for the quantitative determination of PHB and PLA in soil. This technique is based on the analysis of the masses of the products developed during gaseous pyrolysis in an inert atmosphere. A portion of gas degradation products is transferred to a quadrupole mass spectrometer via a heated capillary. The loss of mass at a specific temperature may be related to the mass signal of the gaseous pyrolysis products. These pyrolysis products’ formation can be related to their specific degradation temperatures determined by the TGA. The advantages of the method are the absence of sample pre-treatment and the use of an internal standard but the sample to be measured is often of some micrograms giving problems of homogeneity and significance in sampling (dslu).

#### 4.2.3 High-performance liquid chromatography-HPLC

HPLC is a valid method in the analysis of the soluble fraction of biopolymer degradation products. In the case of PHB analysis the degradation products such as crotonic acid and 2-pentenoic acid, are easily separable and can be quantified. The analytical results are comparable to those obtained with GC-MS, but with shorter analysis times (Duvigneau 2021) and easier sample treatment.

## 5 Remarks

**SCHEME 1:**
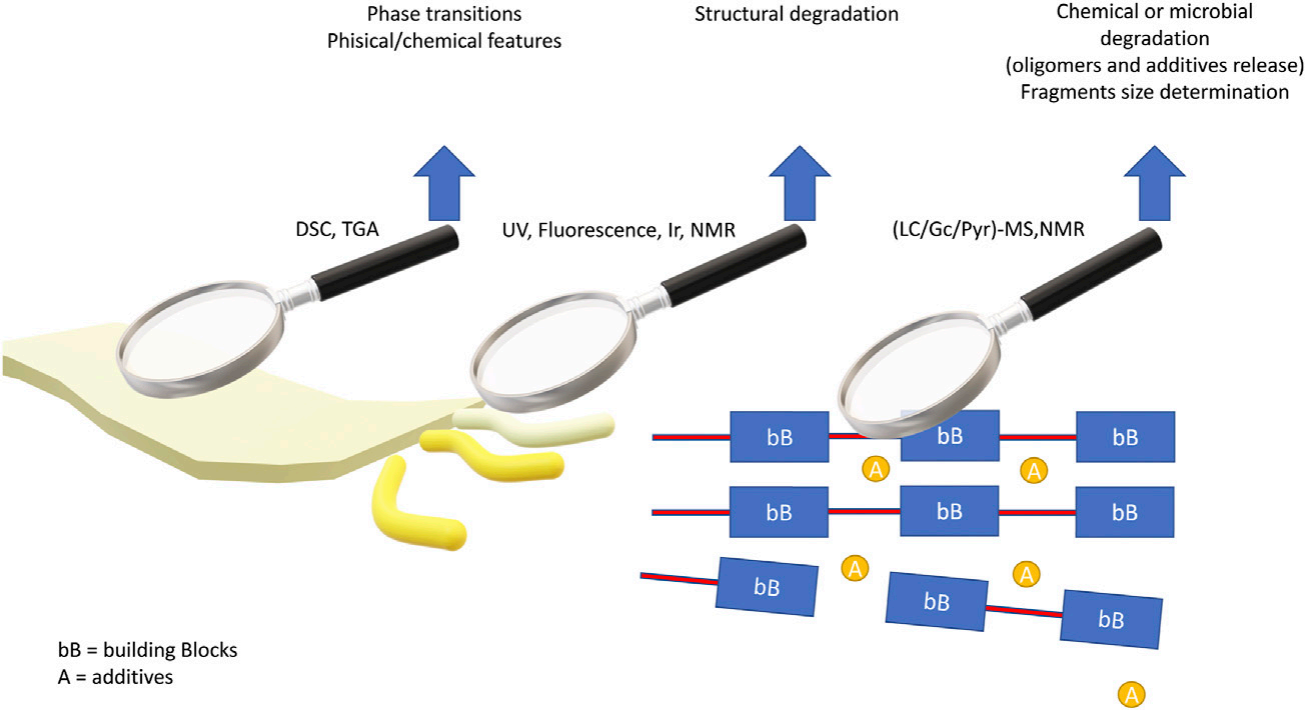
Experimental techniques for the study of (bio) plastic materials.

A wide variety of bioplastics such as PHA, PHB, and PLA have been introduced to address the environmental challenges associated with conventional petroleum-derived plastics. However, also bioplastics have some shortcomings. The information shows that even bio-based plastics cannot be easily recycled. Therefore, bioplastics are also collected in landfills, wherein gradually undergo degradation, leading to CO_2_ and methane formation. Nevertheless, it is important to assess the environmental impact of bioplastics compared to the damage caused by conventional plastics. It is clear that, now, the side effects associated with bioplastics are less serious than that associated with conventional plastics. A critical point concerns the lack of analytical methodologies to determine in aquatic ecosystems the concentrations of soluble fractions resulting from the degradation of biopolymers. According to the literature (Scheme 1) the available quantitative analytical techniques, i.e., HPLC, can measure the concentration of some degradation products under controlled laboratory test conditions, far from the environmental conditions of aquatic ecosystems.

So, it becomes increasingly necessary to develop analytical methods and procedures capable of monitoring these substances in real environments. Since bioplastics are a subject of current interest and scientific study, it is also plausible that their disadvantages can be overcome in the future. Kim et al., 2009.
